# Sensory processing sensitivity and culturally modified resilience education: Differential susceptibility in Japanese adolescents

**DOI:** 10.1371/journal.pone.0239002

**Published:** 2020-09-14

**Authors:** Chieko Kibe, Miki Suzuki, Mari Hirano, Ilona Boniwell

**Affiliations:** 1 Institute for Education and Human Development, Ochanomizu University, Bunkyo-ku, Tokyo, Japan; 2 Institute of Education, Ikubunkan Yume Gakuen, Bunkyo-ku, Tokyo, Japan; 3 Faculty of Humanities, Tokyo Kasei University, Itabashi-ku, Tokyo, Japan; 4 School of Psychology and Sport Science, Anglia Ruskin University, Cambridge, United Kingdom; Chiba Daigaku, JAPAN

## Abstract

This study investigated the efficacy of a culturally modified resilience education program on Japanese adolescents’ well-being from a differential susceptibility perspective. First, a culturally modified resilience education intervention was developed by employing the SPARK resilience program and implemented with 407 Japanese high school students in Tokyo (age = 15–16, M = 192, F = 215). To test intervention efficacy, students’ level of resilience, self-esteem, self-efficacy, and depression were measured pre-, post-, and three months after intervention. Additionally, sensory processing sensitivity, using the Japanese version of the Highly Sensitive Child Scale for Adolescence, was measured as an index of individual sensitivity. Analysis of variance was used to examine the baseline differences and interaction effects of students’ gender and level of sensory processing sensitivity. Latent growth curve models were used to assess the overall effects of the intervention and change over time. Results indicated that the intervention was effective in enhancing students’ overall self-efficacy; and that highly sensitive students, who scored significantly lower in well-being than their counterparts at baseline, responded more positively to the intervention, and had a greater reduction in depression and promotion of self-esteem. These findings provided unique evidence in line with the differential susceptibility perspective and useful implications to develop personalized treatment interventions for adolescents in different cultural contexts.

## Introduction

Mental health problems in the youth population are prevalent across the globe. According to the World Health Organization (WHO), depression is the second-leading cause of illness and disability among young people aged 15–19 years [1]. The symptoms of depression range from cognitive, emotional, motivational, and physical aspects [2]; it interferes with the normal functioning of affected people and its effects encompass disruption of educational attainment, role transitions, and employment stability [3–5]. In Japan, one study found that 7.8% of primary school students and 22.8% of secondary school students showed high levels of depressive symptoms [6]. Also, it was reported that about 3.7% (estimated as 1 in 27) of secondary students were on long-term absenteeism (more than 30 school days) in 2018, which was associated with socio-emotional difficulties that precluded them from attending school [7]. Though the etiology of depression is multifaceted, research suggests that depression has strong relationships with low self-efficacy as well as low self-esteem [8–10]. For instance, while self-efficacy refers to individuals’ beliefs in their own effectiveness that is associated with motivation [11], self-esteem represents individuals’ sense of self-worth [12]. Depressed individuals show symptoms of loss of interest and pleasure in almost all activities and experience feelings of worthlessness with excessive self-blame [2], which imply significant decline in self-efficacy as well as damaged self-esteem. Taken together, efforts to cultivate and enhance positive self-regard (i.e., self-efficacy, self-esteem) are crucial in order to prevent undesirable consequences of mental health problems in Japanese adolescents.

While the family provides a proximal context for fostering positive adolescent self-regard, the school offers an ideal social opportunity through the implementation of evidence-based socio-emotional programs (i.e., universal approach). These endeavors are particularly evident in Western countries, where large-scale approaches have been undertaken [13, 14]. For example, intervention programs that incorporate cognitive behavioral therapy (CBT) are well documented and are often referred to as “resilience programs” as they aim to enhance individual protective factors that prevent negative consequences from life challenges [15]. Among various protective factors, sense of self-worth, self-regulation skills, self-efficacy, and close relationships have been found to contribute to individual resilience in the children’s adaptive systems [16, 17]. While the concept of resilience in developmental sciences refers to “a dynamic process wherein individuals display positive adaptation despite experiences of significant adversity” [18, p. 858], the findings of protective factors that buffer negative influences have greatly informed practical application and helped to shape many preventative interventions. Indeed, research have found that such resilience intervention programs were effective in alleviating mental health problems [14, 19].

However, research also indicates heterogeneous outcomes regarding interventions, depending on participants’ ethnicity or culture, the forms of delivery, and the facilitators [20–22]; furthermore, overall effect sizes were reported to be relatively small [21, 23]. Hence, some issues arise when introducing preventative interventions to Japanese adolescents. First, the feasibility within the cultural context must be considered. Since mental health issues are defined with reference to the socio-cultural background [2], the content of the program must be well-matched with their unique context. One apparent characteristic of the Japanese context is the collectivist culture with high homogeneity, which fundamentally determines individual experiences regarding emotion, cognition, and motivation [24, 25]. Unlike schools in other developed countries (e.g., the United States of America, United Kingdom), little diversity is observed in Japanese high schools. For example, there were less than 0.1% (3,000 out of 3,300,000) of students whose first language was not Japanese among the total high school students in 2016 [26]. This means the vast majority of Japanese high schools consist of solely Japanese nationals. Furthermore, a cross cultural study suggested that emotional aspects of well-being in Japan were closely related to interdependent and interpersonal engagement of the self, whereas Americans were related to independent and interpersonal disengagement of the self [27]. Therefore, when introducing an intervention program for Japanese youth, these contextual differences including emotion, self-regard, and motivational inclination need to be considered. Second, the implementation of the program should be supported by sound empirical evidence. Because the introduction of any new program in a school setting would require considerable investment, the effectiveness of the intervention should be estimated prior to its large-scale application. Thus, potential moderators that would affect the outcomes of a universal approach should be carefully investigated.

Among many factors, gender is a strong predictor of mental health problems and it would interact with intervention outcomes. In particular, adolescent girls are known to be at a higher risk, and gender differences are reported to begin at puberty [28]. Hence, investigating the moderating effect of gender differences would lend valuable insight into implementing preventative intervention for adolescents. In addition, individual personality differences may considerably interact with intervention outcomes. For example, previous research found a strong correlation between depression and neuroticism [29, 30] and suggests that personality differences predict treatment outcomes [30, 31]. Likewise, considering the underlying mechanisms in individual psychobiological bases, namely, sensory processing sensitivity (SPS), would provide useful insight. Research indicates that SPS lays the foundation for registering and processing external information and is associated with a low threshold for environmental stimuli, strong emotional responsiveness, and deep information processing [32, 33]. Individuals with high SPS may also be described as “introverted” in personality and “difficult” in temperament; however, this is conceptually distinct from previously studied personality sub-traits [34, 35]. Moreover, this individual sensitivity—or, more specifically, “susceptibility” to environmental stimuli—has been found to moderate outcomes of person x environmental interactions [36, 37]. This is because susceptible individuals tend to be more responsive to both positive and negative external stimuli; therefore, what are traditionally viewed as vulnerability factors can function as plasticity factors as well [38, 39]. This view has been supported by growing evidence [40, 41] and was theorized as the differential susceptibility theory (DST) from evolutionary–neurodevelopmental perspectives [38, 39, 42]. Employing the DST perspective within a Japanese context, adolescents with high SPS, particularly girls, may report lower well-being and higher levels of depression than less sensitive youths under stressful circumstances; yet, at the same time, they would benefit more from supportive intervention than their counterparts would. Thus, considering students’ gender and SPS as potential moderators would allow for valuable insight into school-based adolescent interventions.

As detailed above, this study aims to address two main issues regarding the implementation of preventative intervention within a Japanese context: 1) the intervention program should be culturally sensitive, and thus, modifications must be made when applying it to Japanese adolescents; 2) intervention outcomes should be adequately estimated, as it is likely that students’ gender and susceptibility would moderate intervention outcomes. Therefore, in an attempt to facilitate effective intervention in the Japanese context, this study developed a Japanese version of intervention by employing a proven program and sought to test its overall effectiveness with respect to students’ well-being. Then, to investigate the moderation effects of gender and individual sensitivity from a DST perspective, we hypothesized that highly sensitive youths, particularly girls, would report lower well-being and higher depression at baseline but would show greater positive outcomes than less sensitive students after intervention. Since few empirical studies on this subject have been reported in Japan, or more broadly, non-Western countries [43], this empirical study will provide a valuable contribution to adolescent intervention studies in different cultural contexts and will further our understanding of person x environmental interactions.

## Materials and methods

### Development of the intervention program

First, in an attempt to develop a culturally suitable intervention, we employed an existing intervention program: the SPARK resilience program, which was originally developed and validated in the United Kingdom [36, 44, 45]. This program was developed based on CBT components and incorporating findings from resilience studies, which aims to foster protective factors (e.g., self-esteem, self-efficacy, self-regulation skills) to promote individual resilience and prevent depression [44, 45]. By employing this intervention program, we first created a translated version of the program in collaboration with bilingual professionals; then consulted with school teachers and a school psychologist, who had extensive experience in clinical work, about its applicability to Japanese adolescents. Through the course of consultation, three major issues were raised: 1) the time constraints within school curricula to implement a whole program, 2) the relevance of the examples and case studies, and 3) the order of lesson delivery in consideration of students’ acceptability. To resolve the first two issues, we carefully modified the time allocation of lessons by compressing the original 12 one-hour package to a 6 one-hour package and replaced case studies with more familiar examples. Although the reduction in the lesson hours might have affected the efficacy of the program, it would not have otherwise been possible to implement it in the existing school curricula. Therefore, we prioritized the pragmatic accommodation by ensuring the program quality. The last issue concerned not only practical aspects of lesson delivery, but also acceptability, and by extension, the efficacy of the program. The original program was designed to introduce the roles of, and the ways to deal with one’s cognition, and then proceed to deal with emotions. However, as adolescence is a phase marked with heightened emotionality [46] and the interdependent nature of the Japanese self-construct would likely make them more susceptible to others’ emotions rather than their own cognition [27], we modified the lesson order to first introduce and deal with emotions, then proceed to cognition. The modification process was carefully discussed with relevant professionals, practitioners, as well as the original program developers. Table 1 shows the Japanese version of the program and its comparison with the original program.

**Table 1:** Japanese version of the SPARK resilience program with reference to the UK version.

	Lesson	Contents	UK Lesson
1	What is resilience?	Introduction of multidimensional construct of resilience	1
2	Magic of distraction	Role of emotions and skills to deal with them	8
3	Resilience muscle training	Protective factors enhancing resilience	11
4	Growing from adversity	Concepts and examples of post-traumatic growth	10
5	Understand the negative spiral	Psychological mechanism of negative spiral (CBT model)	2, 3, 4, 5
6	Challenge your negative spiral	Role of perception and challenge in the negative spiral	6, 7, 9

Each lesson consists of interactive lectures and practical activities. The original lesson plan can be found in [44, 45].

### Intervention procedure

#### Study context and participants

The modified version of the SPARK resilience program was introduced to the first-grade students (aged 15–16 years) of a high school in Tokyo. The selected school has a unique curriculum in that it sends all second graders for a one-year overseas program with the aim to develop their English proficiency through real life experiences. According to the Tokyo Metropolitan Government, the average tuition fee for the first year of the private high school is JPY934,000 (approximately US$8,780), whereas the government school’s fee for the first year is JPY125,000 (US$1,176) [47, 48]. Additionally, sending the students to the one-year overseas program requires substantial cost for the family, which indicates that parents or caregivers of students of the current school are high income families. Though the number of students with special educational/medical needs is quite low in this school with an average of around 3% each year, there is a school psychologist who closely monitors their school adjustment.

While this overseas program has benefitted students’ development in many ways, a certain number of students developed mental health problems and had to terminate their participation in the program. An informal survey conducted by the school psychologist in the previous year indicated that the students exhibited high level of anxiety before the overseas program. Therefore, the teachers wished to enhance students’ resilience (i.e., protective) factors to promote their well-being, and consulted the school psychologist, who had regular contact with the students, to provide preventative measures before the students’ departure. Consequently, the program was introduced as a universal educational intervention within regular lessons in a span of three months in the first year of high school; that is, one year prior to their participation in the program. Before the program implementation, the school psychologist was trained on the program, and the school’s deciding committee led by the school principal agreed to provide the intervention program by the aforementioned school psychologist consistently, and the board waived the need for parental consent. The students answered self-report questionnaires pre- and post-intervention, as well as three months afterward. When administering the pre-intervention questionnaire at the beginning of the academic year, the students were informed of the purpose of the program and the survey, and their right to withdraw their participation from the study; their verbal consent was obtained and recorded on the register list.

**Fig 1:**
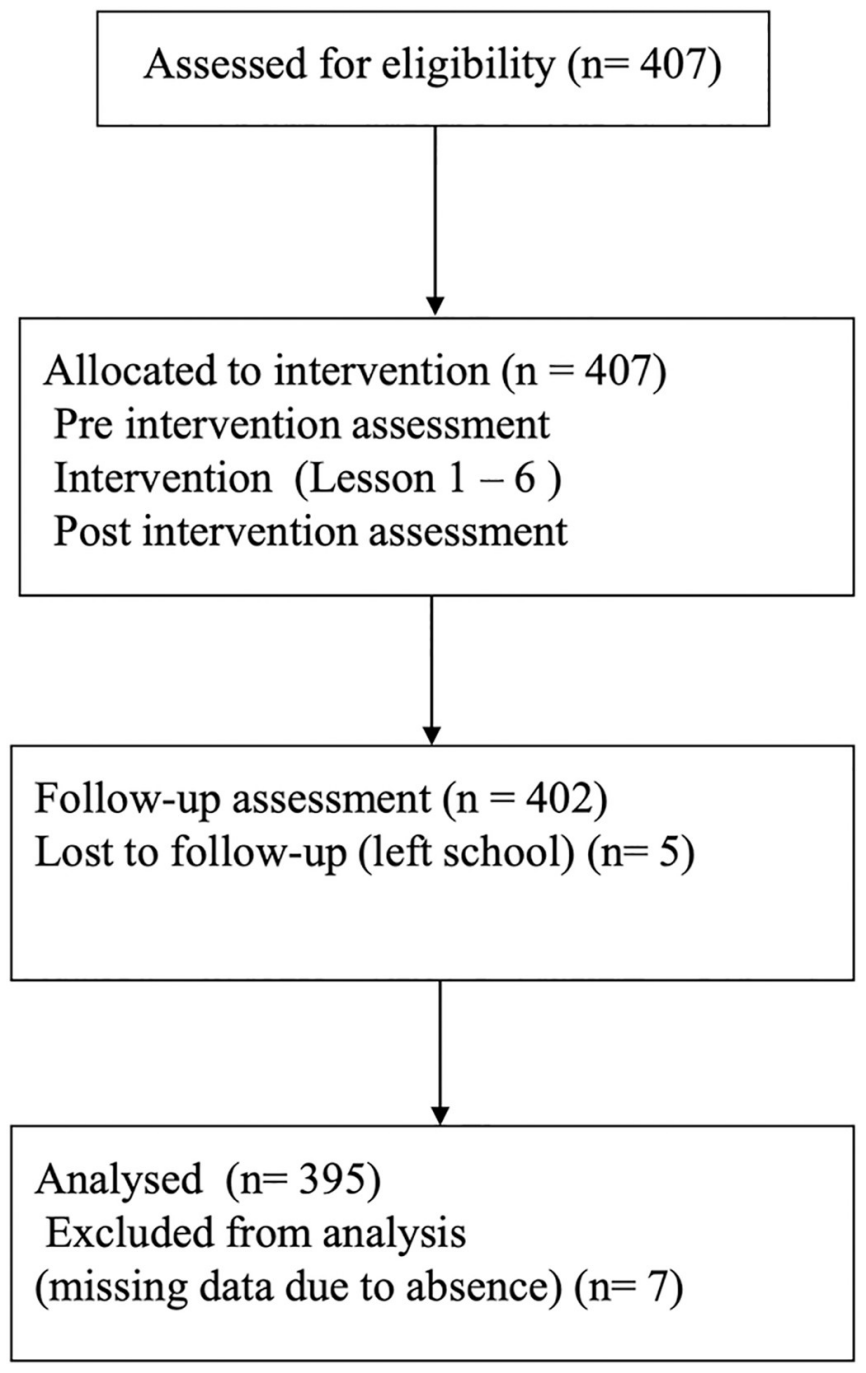
Flowchart of the resilience education intervention.

This psychoeducational support has been carried out by the same school psychologist for all first graders from three classes (with the typical class size of 45 students) since 2015. While no compensation was given to the students for participating in the intervention, the students received their own “resilience album” upon completion of the intervention each year, and most of them took these albums to their overseas programs. To maximize the statistical power, and to address the current research purposes, we used aggregated data from three cohorts who received the above mentioned intervention (*N* = 407, M = 192, F = 215). We confirmed no statistical differences among the study variables in these three cohorts. As shown in Fig 1, the final data consist of 395 (M = 174, F = 221) because some students left school amid term or absent on the days of the session, hence, were unable to complete the questionnaire. We also confirmed there was no statistical differences between the participants who completed the program and those who dropped out. The institutional review board of Tokyo Kasei University approved this study based on the agreement from the school board (H30-08). The study protocol and detailed lesson plans can be found in supporting materials (S1–S5 Files).

### Outcome measures

As a measure of students’ self-regard, self-esteem was measured using the Rosenberg Self-Esteem Scale [49, 50]. This is a 10-item questionnaire with a 4-point scale ranging from 1 (strongly disagree) to 4 (strongly agree), which asks about the respondent’s evaluation of self-worth. The total score was calculated; higher scores represented positive self-regard. Additionally, self-efficacy was measured using the General Self-Efficacy Scale [51, 52]. This is a 10-item questionnaire that measures a respondent’s general self-efficacy on a 4-point scale ranging from 1 (strongly disagree) to 4 (strongly agree). The Cronbach’s alpha of each scale was excellent (α = .80 and .90 for self-esteem and self-efficacy, respectively).

In this study, we also measured students’ perception of resilience factors using the Bidimensional Resilience Scale [53], which was developed and validated in Japan. This is a 21-item measure with a 5-point scale ranging from 1 (strongly disagree) to 5 (strongly agree). Although it has seven subscales under two dimensions (i.e., innate and acquired), we used the mean score of the 21 items to represent individual resilience levels. The English translation of this scale can be found in the supporting materials (S6 File). The Cronbach’s alpha of this scale was excellent (α = .87).

To test the intervention efficacy with respect to mental health prevention, we measured students’ depression levels using the Birleson Depression Self-Rating Scale for Children [54, 55]. This is an 18-item measure with a 3-point scale ranging from 0 (never) to 2 (most of the time), and it evaluates the mood, cognitive, and somatic aspects of depressive symptoms. The total score was used as an index of students’ depression levels. The Cronbach’s alpha of this scale was also excellent (α = .86).

In addition, to test the moderation effects of an individuals’ susceptibility, we measured students’ SPS using the Japanese version of the Highly Sensitive Child Scale for Adolescence (J-HSCS) [56]. This scale is a translated version of the Highly Sensitive Child Scale [33], originally developed and validated in the United Kingdom. Although the original English version has 12 items, the J-HSCS consists of 11 items, as one of them did not yield a sufficient factor loading [56]. While the J-HSCS was reported to have three subscales (aesthetic sensitivity, low sensory threshold, and ease of excitation), the purpose of this study was to evaluate the overall adolescents’ sensitivity; therefore, we calculated the mean score of 11 items and then created the study variables. The Cronbach’s alpha of this scale was acceptable (α = .70).

### Analysis plan

**Fig 2:**
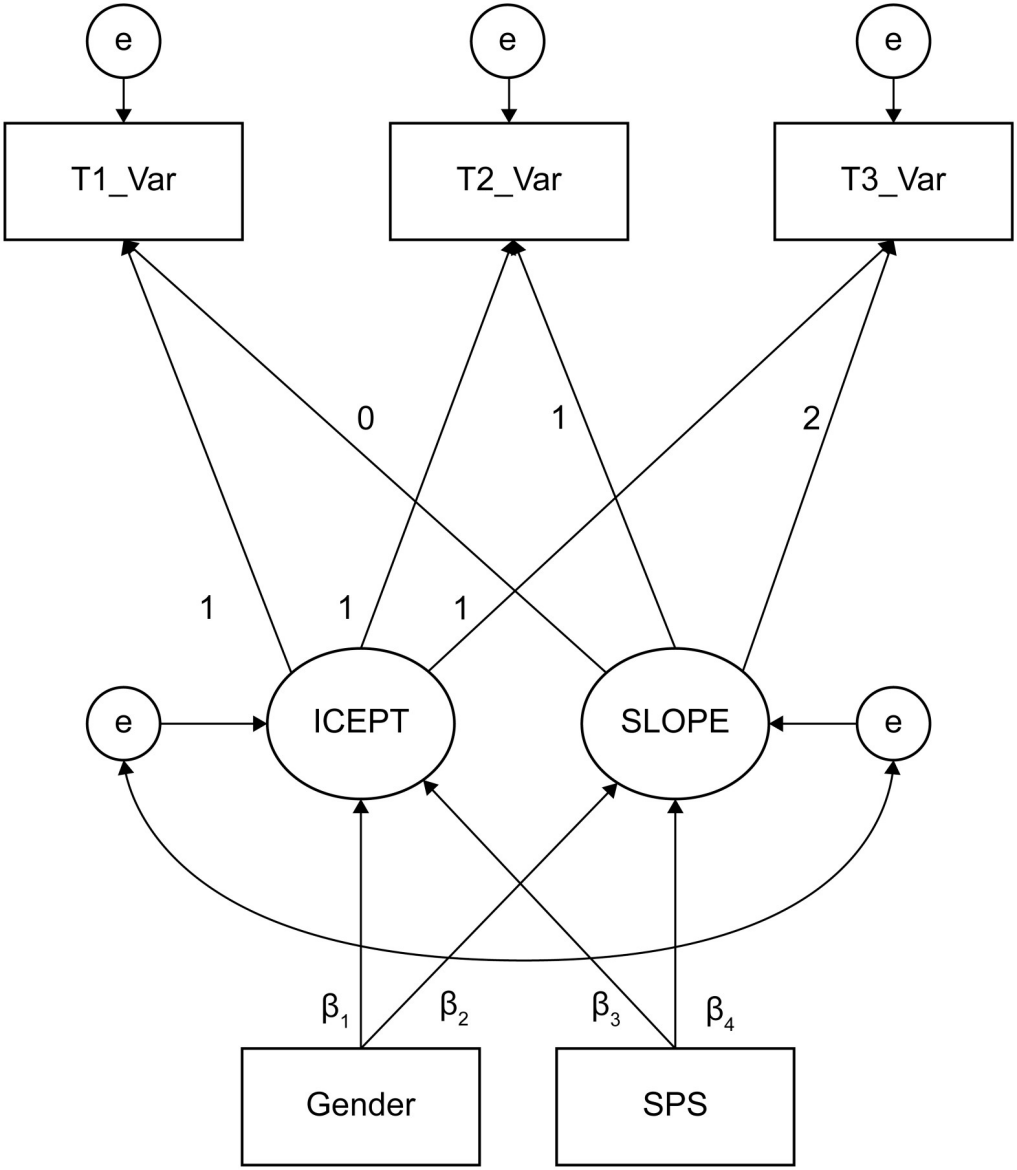
Hypothesized latent growth curve model with gender and SPS as predictor variables. T1, T2, and T3 represent pre-, post-, and three-months follow-up measurements, respectively. “Var” indicates study variables measured with the Bidimensional Resilience Scale (BRS), Rosenberg Self Esteem Scale (RSES), General Self Efficacy Scale (GSES), and the Birleson Depression Self Rating Scale for Children (DSRS). SPS was measured with the Japanese version of the Highly Sensitive Child Scale for Adolescence (J-HSCS).

As a first step, we examined the descriptive data and bivariate correlation among the study variables. Further, to test the overall effects of the culturally modified intervention, we conducted a latent growth curve model (LGCM) analysis [57] on all the students’ data and assessed their changes from pre- to post-intervention, as well as three months after the intervention. The LGCM is an excellent approach that allows us to see the group mean and individual variance at baseline (i.e., intercept) and the rate of change over time (i.e., slope). After examining the overall effects, we conducted a two-way analysis of variance (ANOVA) by creating two gender groups and three SPS groups (i.e., high [+1*SD*], middle, and low [−1*SD*] on the J-HSCS) to examine the baseline differences and interaction effects of students’ gender and level of SPS. Finally, we conducted further LGCM analysis with gender and individual SPS as predictors of the model. Fig 2 illustrates the hypothesized model of the final LGCM. In this model, if the estimated regression weight from the predictors to the intercept (i.e., β_1_, β_3_) was found to be significant, it would indicate a significant baseline difference as a function of the predictors. Likewise, if the coefficients from the predictors to the slope (i.e., β_2_, β_4_) were found to be significant, it would suggest that the changes after the intervention were moderated by a function of the predictors. In this study, all analyses were conducted using SPSS (ver. 23) and Amos (ver. 23), and the level of significance was set at α = .05.

## Results

Table 2 presents the descriptive statistics and bivariate correlation among the study variables. As previous studies have indicated, self-esteem and self-efficacy showed a significant negative association with students’ depression, cross-sectionally and longitudinally. Additionally, students’ resilience score was found to have a negative association with depression and a positive association with self-esteem and self-efficacy. Interestingly, a higher level of sensitivity showed a negative association with self-esteem and self-efficacy and a positive association with depression.

**Table 2:** Summary of intercorrelations, means, and standard deviations for main variables.

		1	2	3	4	5	6	7	8	9	10	11	12	13	*M*	*SD*
1	T1_J-HSCS														5.08	0.80
2	T1_BRS	-.06													74.45	12.17
3	T2_BRS	-.06	.59***												75.37	12.32
4	T3_BRS	-.02	.54***	.75**											74.42	13.56
5	T1_RSES	-.29***	.53***	.42**	.40**										24.56	5.76
6	T2_RSES	-.15**	.32***	.51***	.44***	.55***									25.28	5.71
7	T3_RSES	-.13**	.29***	.47***	.52***	.53***	.70***								25.07	5.53
8	T1_GSES	-.13*	.71***	.47***	.40***	.60***	.39***	.35***							26.68	5.65
9	T2_GSES	-.11*	.49***	.69***	.57***	.43***	.59***	.45***	.56***						27.38	5.84
10	T3_GSES	-.04	.42***	.57***	.71***	.42***	.47***	.60***	.48***	.56***					27.66	6.51
11	T1_DSRS	.27***	-.52***	-.35***	-.38***	-.59***	-.36***	-.39***	-.45***	-.34***	-.36***				11.70	6.26
12	T2_DSRS	.22***	-.37***	-.52***	-.47***	-.42***	-.57***	-.53***	-.34***	-.48***	-.38***	.60***			11.36	6.33
13	T3_DSRS	.12*	-.31***	-.43***	-.56***	-.38***	-.45***	-.62***	-.24***	-.35***	-.48***	.58***	.68***		11.90	6.66

*p< .05,

**p< .01,

***p< .001.

Intercorrelations of study variables for all participants (*N* = 407) are presented. Scale abbreviations are: J-HSCS = the Japanese version of Highly Sensitive Child Scale for Adolescence, BRS = Bidimensional Resilience Scale, RSES = Rosenberg Self Esteem Scale, GSES = General Self Efficacy Scale, and DSRS = the Birleson Depression Self Rating Scale for Children.

The results of the LGCM analysis to examine the overall intervention effects on the study variables are presented in Table 3. Results indicated that the intervention positively affected the enhancement of students’ overall self-efficacy, with an excellent model fit (*x*^*2*^ = .66, *p* = .42, comparative fit index [CFI] = 1.00, root mean square of approximation [RMSEA] = .00). Notably, the variance of the intercept was statistically significant, which indicates that the inter individual differences were large at baseline; additionally, the mean level of change (i.e., slope) was statistically positive (β = .48, *p* < .001), suggesting an overall positive effect of the intervention in self-efficacy enhancement. However, except for the above mentioned effects, no statistical difference was detected before and after the intervention regarding resilience, self-esteem, or depression.

**Table 3:** Efficacy of the intervention in latent growth curve model (LGCM).

	Intercept		Slope		Fit Indices			
	*M*	*Var*	*M*	*Var*	*x*^*2*^ *(df)*	*p*	CFI	RMSEA
BRS	74.69***	88.91***	-0.09	18.26***	4.21 (1)	.04	.99	.09
	(-0.58)	(11.66)	(0.31)	(5.69)				
RSES	24.72***	19.72***	0.18	3.47***	3.34 (1)	.07	.99	.08
	(0.28)	(2.71)	(0.14)	(1.18)				
GSES	26.75***	19.17***	0.48***	2.12	0.66 (1)	.42	1.00	.00
	(0.28)	(2.97)	(0.16)	(1.48)				
DSRS	11.58***	22.50***	0.14	1.57	3.96 (1)	.05	.99	.09
	(0.30)	(3.26)	(0.15)	(1.56)				

***p< .001. *M* represents overall mean effect on the study variables, while *Var* represents variance of individual differences in the study variables. CFI = comparative fit index; RMSEA = root mean square of approximation; BRS = Bidimensional Resilience Scale; RSES = Rosenberg Self Esteem Scale; GSES = General Self Efficacy Scale; and DSRS = the Birleson Depression Self Rating Scale for Children. The numbers in the parenthesis are standard errors for means and variance of each variable.

Having examined the overall efficacy of the intervention, we proceeded to investigate the moderation effects of individual differences. First, we investigated baseline differences with a two-way ANOVA based on students’ gender and sensitivity groups (Table 4). The results found a main effect of gender on self-esteem, which indicated that female students tended to report lower self-esteem (*F*(1, 391) = 3.94, *p* < .05). Also, main effects of sensitivity difference were found with self-esteem (*F*(2, 391) = 8.26, *p* < .001), self-efficacy (*F*(2, 389) = 2.43, *p* < .01), and depression (*F*(2, 388) = 11.00, *p* < .001); these results confirmed that highly sensitive adolescents, both male and female, reported a significantly lower level of self-esteem and self-efficacy and a higher level of depression. Additionally, the results indicated interaction effects between gender and sensitivity on resilience (*F*(2, 394) = 3.63, *p* < .05), indicating that highly sensitive male students reported significantly lower levels of resilience at baseline. In sum, these results partially supported our hypothesis that girls and highly susceptible individuals would report lower levels of well-being without adequate support (i.e., at baseline).

**Table 4:** Baseline differences for main variables by gender, SPS, and interactions.

		Male		Female		Main effects (Gender)	Main effects (SPS)	Interaction effects (Gender x SPS)
		*M*	*SD*	*M*	*SD*	*F(df)*	*F(df)*	*F(df)*
BRS	SPS (0)	73.53	14.45	76.23	13.28	0.41 (1, 394) *n*.*s*.	1.45 (2, 394) *n*.*s*.	3.63 (2, 394) *p*< .05
	SPS (1)	76.87	10.57	73.26	11.63			
	SPS (2)	70.92	13.92	74.26	10.54			
RSES	SPS (0)	27.08	4.94	25.75	5.66	3.94 (1, 391) *p*< .05	8.26 (2, 391) *p*< .001	0.40 (2, 391) *n*.*s*.
	SPS (1)	25.22	5.51	23.47	5.37			
	SPS (2)	23.50	6.67	23.03	5.97			
GSES	SPS (0)	27.43	5.62	27.85	6.32	0.14 (1, 389) *n*.*s*.	2.43 (2, 389) *p*< .01	1.46 (2, 389) *n*.*s*.
	SPS (1)	27.32	5.35	25.78	4.93			
	SPS (2)	25.72	5.58	26.17	6.24			
DSRS	SPS (0)	9.25	5.71	10.11	5.76	1.00 (1, 388) *n*.*s*.	11.00 (2, 388) *p <* .001	0.04 (2, 388) *n*.*s*.
	SPS (1)	11.39	6.18	11.84	5.92			
	SPS (2)	13.38	7.04	14.01	6.28			

SPS (0), (1), (2) each represents low (-1*SD*), medium, high (+1*SD*) in SPS group respectively. SPS was measured with the Japanese version of Highly Sensitive Child Scale for Adolescence (J-HSCS). BRS = Bidimensional Resilience Scale; RSES = Rosenberg Self Esteem Scale; GSES = General Self Efficacy Scale; and DSRS = the Birleson Depression Self Rating Scale for Children.

Finally, to test the moderation effects of the predictors, we ran an LGCM analysis on each study variable (Table 5). The results indicated moderation effects on depression fit best with the current data, yielding excellent model fit (*x*^*2*^ = 7.40, *p* = .19, CFI = 1.00, RMSEA = .03). In this model, a significant regression weight from the SPS predictor to the intercept (β = 2.05, *p* < .001) and slope (β = −.51, *p* < .01) were found, which suggested that highly sensitive students reported higher levels of depression at baseline; yet, after the intervention, they reported a larger rate of positive change. Nevertheless, such moderation effects were not found with respect to gender. While we could not find convincing moderation effects on other variables, there was a good indication of a similar trend in self-esteem changes (intercept: β = −1.88, *p* < .001; slope: β = .60, *p* < .001; *x*^*2*^ = 29.47, *p* = .00, CFI = .95, RMSEA = .11). Therefore, to further investigate the moderation effect, we conducted a post-hoc analysis by examining each predictor one at a time; that is, rather than testing two predictors simultaneously, we ran an LGCM analysis with a single predictor. Additionally, we freed one parameter estimate from the slope to the T3_Var [57]. The results showed improvement of the model with the SPS predictor, as indicated with the Akaike information criterion (AIC) change from 59.47 to 23.93, and it yielded excellent model fit (*x*^*2*^ = 1.93, *p* = .59, Δ*x*^*2*^ = 27.53, CFI = 1.00, RMSEA = .00) with the regression weight on the intercept β = −2.10 (*p* < .001) and the slope β = 1.15 (*p* < .001). Although the LGCM with the gender predictor improved the model fit as well (the AIC changed from 59.47 to 24.94; *x*^*2*^ = 2.94, *p* = .40, Δ*x*^*2*^ = 26.53, CFI = 1.00, RMSEA = .00), the moderation effect was observed only with the intercept (β = −1.48, *p* < .001) but not the slope (β = .50, *n*.*s*.). These post-hoc results suggested that both male and female adolescents with higher sensitivity reported lower levels of self-esteem at baseline, however, these highly sensitive adolescents showed larger improvements in self-esteem scores after the intervention program. These findings provided supportive evidence for our hypothesis that highly sensitive individuals tend to report lower well-being at baseline, reflecting their high susceptibility. Nonetheless, they benefit more from the intervention, with larger plasticity, which reflects differential susceptibility perspectives.

**Table 5:** Effects of gender and SPS as predictors on efficacy of the intervention in latent growth curve model (LGCM).

	Predictors	Intercept		Slope		Fit Indices			
		β	*SE*	β	*SE*	*x*^*2*^ *(df)*	*p*	CFI	RMSEA
BRS									
	Gender	0.19	1.18	0.63	0.64	17.41 (5)	.00	.97	.08
	SPS	-0.93	0.74	0.29	0.40				
RSES									
	Gender	1.04	0.54	-0.08	0.28	29.47 (5)	.00	.95	.11
	SPS	-1.88***	0.34	0.60***	0.17				
GSES									
	Gender	0.48	0.55	0.15	0.32	401.46 (5)	.00	.00	.44
	SPS	-0.93**	0.35	0.29	0.20				
DSRS									
	Gender	-0.45	0.59	-0.10	0.30	7.40 (5)	.19	1.00	.03
	SPS	2.05***	0.37	-0.51**	0.19				

**p< .01,

***p< .001.

β represents estimated regression weight of predictors. SPS was measured with the Japanese version of Highly Sensitive Child Scale for Adolescence (J-HSCS).

CFI = comparative fit index; RMSEA = root mean square of approximation; BRS = Bidimensional Resilience Scale; RSES = Rosenberg Self Esteem Scale; GSES = General Self Efficacy Scale; and DSRS = the Birleson Depression Self Rating Scale for Children.

## Discussion

The purpose of the current study was twofold: 1) to develop a culturally suitable preventative intervention and evaluate its overall efficacy; and 2) to investigate the moderation effects of gender and SPS on Japanese youths’ well-being from a DST perspective. To pursue the latter objectives, we hypothesized that highly sensitive individuals, particularly girls, would be at a higher risk of mental health problems, and hence, they would report lower levels of self-regard and higher levels of depression at baseline. Moreover, by employing DST perspectives [38, 39, 42], we also hypothesized that these same—seemingly vulnerable—individuals would benefit more from supportive intervention.

Our first purpose to develop a culturally modified intervention was achieved through the collaboration of multiple professionals and practitioners. The informal feedback from the students and the teachers were overall positive, which may partly owe to the experienced facilitator’s delivery. Yet, the result from the overall evaluation utilizing an LGCM analysis yielded small but significant intervention effects on the enhancement of adolescents’ general self-efficacy, irrespective of gender and individual SPS differences. The outcomes of this intervention appear to be in line with previous findings from studies on school-based Social and Emotional Learning (SEL). Results of a meta-analysis also indicated significant effects in enhancing students’ attitudes toward self (e.g., self-efficacy) [58]. The program modification in this study, which dealt with emotions prior to cognition aiming to suit Japanese students, might have favorably impacted the outcome as well. According to Bandura’s proposition [11], the expectations of personal efficacy are derived from four major principal sources—individual performance accomplishments, vicarious experiences, exhortation, and physiological state. In particular, the fourth component of physiological status refers to emotional constituents, such as relaxation and desensitization, which would ease personal emotional burdens (e.g., fear, anxiety) to undertake target behavior. Given the unique context of the study participants, attending a one-year overseas program would add tremendous pressure. Acknowledging and learning skills to deal with emotions might have contributed to build a sense of self-reliance, thus promoting self-efficacy. Nonetheless, we could not find beneficial effects on the reduction of depression, nor the enhancement of other study measures (i.e., self-esteem, resilience). These null outcomes seem to be inconsistent with the findings of studies conducted with western adolescents [19, 23]. However, the findings somehow resonate with previous findings that pointed out the heterogeneous effects of interventions [20, 21].

Therefore, our next step of inquiry examined the interaction effects of moderators that might have masked the effectiveness of the intervention. First, we tested baseline differences as a function of gender and SPS differences with a two-way ANOVA. The results found a main effect of gender on self-esteem, indicating that girls tended to report lower self-esteem than boys; however, this gender main effect was not found with other variables. Although research suggests that adolescent girls are at a higher risk of depression [28], the results from the present study did not find such a tendency. Rather, individual SPS showed significant main effects on self-esteem, self-efficacy, and depression; that is, highly sensitive students showed lower self-regard and higher levels of depression. The main effects of youth SPS can be understood from a diathesis–stress framework. Given the context of the present study, the students were to depart for a long-term overseas program, and they might have been dealing with considerable levels of stress and anxiety. When such external stress interacts with dispositional susceptibility, it would negatively affect the students’ psychological well-being (i.e., dual risk), and this might have been pronounced in the baseline differences.

Consequently, the next question was whether the preventative intervention would successfully alleviate these adolescents’ mental health conditions and promote their positive self-regard. We carried out an LGCM analysis with predictor variables to examine the interaction effects of gender and individual SPS. The results indicated significant interaction effects of SPS on the reduction of depression. Highly sensitive adolescents, who initially reported lower well-being, experienced greater benefit from the intervention, and an increase in self-esteem was also observed. As the literature on DST suggests, susceptible individuals are more responsive to both positive and negative external information [38, 39, 42]; thus, this moderation effect can function as a “hidden efficacy” of the interventions [59]. Importantly, few empirical studies have been conducted in non-western contexts [39, 43]; nonetheless, these results replicated previous findings on the moderation effects of SPS on intervention efficacy reported from different cultural backgrounds [36, 37]. This suggests that phenotypic SPS—underpinned by biophysiological substrates [32, 34, 35]—predicts the strength of individual responsiveness to the external information irrespective of the societal differences. In other words, although the mean distribution or population may vary, SPS would manifest common functionality in the context of person and environment interaction even in different cultural contexts. Furthermore, these moderation effects seem to corroborate findings from previous research that indicated target-group interventions were more effective than a universal approach [20, 23]. From a practical point of view, it would be beneficial to inform school professionals about these moderation effects of individual differences in the universal approach, with a scope to optimize intervention efficacy and to ultimately better accommodate effective intervention for young people.

Finally, among the study variables, resilience did not yield either overall effects or moderated effects in the present study. Although it goes beyond our scope to discuss conceptional and operational issues surrounding resilience research in detail [60], it would be feasible to interpret our findings as proof of the complexity of the construct, which requires a longer time frame to ascertain, and the ultimate requirement of interaction with adversity. However, our investigation revealed that highly sensitive boys scored lowest in resilience levels before the intervention. While the present study did not find specific effects on this particular group of students, the question emerged as to whether research attention has traditionally been disproportionately focused on “vulnerable girls.” Perhaps societal expectations (e.g., masculinity) pose more pressure on sensitive boys to meet societal standards, such that it eventually becomes detrimental to their well-being. Nevertheless, future research is needed to identify the problem and investigate the mechanism. Furthermore, this study found favorable effects on susceptible individuals, but not for less susceptible adolescents. Although the definitive mechanism is unclear, this might have been due to ceiling effects. Since the students already showed high level of well-being in the beginning, their change over time could not have been adequately captured with the current measurement. In this regard, a qualitative approach, such as interviews, would help refine the intervention program. Nonetheless, these findings emphasize the need to optimize intervention efficacy according to adolescents’ responsiveness. As mentioned earlier, personality differences have been found to predict depression and long-term outcomes [30, 31]; therefore, clinical treatment is personalized under expert supervision. Given that sensitivity differences interact with intervention efficacy, similar personalized treatment ought to be made available under adequate supervision, even in the case of universal interventions.

This study provided empirical evidence for the efficacy of a culturally modified resilience education and discussed the interaction effects of individual sensitivity from the DST perspective. While it contributes uniquely by providing novel evidence, it also warrants further refinement to overcome limitations. First, the intervention was implemented only in one high school with a single-arm design; therefore, the generalizability of the current findings should be carefully considered. Replication of this study in other school settings, preferably with a control group randomized design, would be necessary. Particularly, as adolescence is a phase of dynamic change, investigating different age groups, in consideration of potential moderation effects, would lend further insight. Additionally, while the intervention aimed to prevent mental health problems, no clinical diagnoses were made; hence, other therapeutic/medical treatment that the students might have received have not been accounted for. Further, the data collection was completed within one year. A follow-up survey over a longer time span would facilitate determining long term outcomes.

## Conclusion

The current paper provided novel evidence of a culturally modified intervention program and its efficacy for promotion of students’ self-efficacy. Though youth mental health problems are prevalent across the globe, studies on culturally suitable interventions are not abundant. The findings of this study indicated the value of accommodating intervention programs according to the target population’s cognitive, emotional, and motivational inclinations. In addition, the findings of moderation effects of SPS highlighted the importance of considering individual differences when implementing a universal approach intervention. These results could be particularly useful when designing personalized interventions for the best interests of the target individuals.

## Supporting information

S1 File TREND checklist. (DOCX)

S2 File Intervention study protocol (Japanese). (DOCX)

S3 File Intervention study protocol (English). (DOCX)

S4 File Lesson plan (Japanese). (DOCX)

S5 File Lesson plan (English). (DOCX)

S6 File Items of the Bidimensional Resilience Scale (BRS). (DOCX)

S7 File Dataset. (SAV)
