# Supplementary material for: Sensory processing sensitivity and culturally modified resilience education: Differential susceptibility in Japanese adolescents
Source: PLoS One. 2020 Sep 14;15(9):e0239002. doi: 10.1371/journal.pone.0239002 (PMC7489542; doi:10.1371/journal.pone.0239002)
Supplement: S2 File — (DOCX) [file pone.0239002.s002.docx]

**S2 File. Intervention Study Protocol (Japanese)**

**レジリエンス教育実践研究の手続き**

本実践研究は，協力校における生徒への心理的支援の一環としてプログラムを行い，その効果を検討することを目的とする。プログラムの実施は校内勤務のスクールカウンセラーが担当し，データ処理，分析などは研究者が行う。具体的な手順は下記の通りである。

1. 心理教育実施前の測定　（4月）

プログラム参加予定の生徒のベースラインアセスメントを実施

1. 心理教育の実施　（6月～9月）

スクールカウンセラーによる心理教育の実施

- 1. レッスン１・２
  2. レッスン３
  3. レッスン４
  4. レッスン５・６

1. 心理教育実施後の測定 （9月）

上記全ての授業終了後に事後測定を行う

1. フォローアップ測定（12月～翌1月）

心理教育実施の3か月後にフォローアップ測定を実施

1. データ分析

収集されたデータは匿名化した後，研究者が分析を行う。
