# Supplementary material for: Sensory processing sensitivity and culturally modified resilience education: Differential susceptibility in Japanese adolescents
Source: PLoS One. 2020 Sep 14;15(9):e0239002. doi: 10.1371/journal.pone.0239002 (PMC7489542; doi:10.1371/journal.pone.0239002)
Supplement: S3 File — (DOCX) [file pone.0239002.s003.docx]

**S3 File. Intervention Study Protocol (English)**

This study aims to provide psychoeducational supports for the students in collaboration with a school, and to investigate the efficacy of the program. While the program is facilitated by a school counselor, the data analyses are undertaken by the researchers. The study protocol is outlined below.

1. Pre intervention assessment (April)

Students take assessment at baseline condition

1. Intervention procedure (June to September)

The following lessons are provided within a school curriculum in a span of three months period. The facilitator is a school counselor.

- 1. Lesson 1, 2
  2. Lesson 3
  3. Lesson 4
  4. Lesson 5, 6

1. Post intervention assessment (September)

Upon completion of the lessons mentioned above, the students are to take post-intervention assessment.

1. Follow up assessment (December/January)

After three months from the completion of the program, the students are to take follow-up assessment.

1. Data analysis

After the completion of the data collection, the data is analyzed anonymously by the researchers.
