# Supplementary material for: Sensory processing sensitivity and culturally modified resilience education: Differential susceptibility in Japanese adolescents
Source: PLoS One. 2020 Sep 14;15(9):e0239002. doi: 10.1371/journal.pone.0239002 (PMC7489542; doi:10.1371/journal.pone.0239002)
Supplement: S4 File — (DOCX) [file pone.0239002.s004.docx]

| **S4 File.　Lesson Plan (Japanese) レジリエンス授業案** | | |
| --- | --- | --- |
| Lesson 1　「レジリエンス」とは何か | | |
| ●授業の目標  ・「レジリエンス」を理解できる  ・「レジリエンス」を定義づけることができる | | 授業で必要なリソース  ・ワークシート  ・詳細は書籍資料セッション1, 2 |
| 導　入  展　開１  展　開２  展　開３  まとめ | 「レジリエンス」の紹介  ・プログラムとレッスンの目的を説明する  ・ネガティブ感情の特徴とネガティブ・スパイラルについて説明する  ・レジリエンスの特徴について紹介する  ワーク1  ・生徒の「レジリエンス」についての理解を促進する   ワーク2  ・生徒が自分の言葉で「レジリエンス」について説明する    ワーク3  ・「レジリエンス」をイメージで例えワークシートに描くよう指示する   まとめと宿題  ・教師によるレッスンのまとめ  　宿題：「レジリエンス」の実例を探すよう指示する | |
| Lesson 2　気晴らしの魔法 | | |
| ●授業の目標  ・気分転換の効果を理解する  ・自分なりの効果的な気分転換の方法を見つける | | 授業で必要なリソース  ・ワークシート  ・詳細は書籍資料セッション3 |
| 導　入  展　開１  展　開２  展　開３  まとめ | 気分転換の大切さの説明  ・L1ネガティブ・スパイラルについて復習する  ・自己の感情認識の大切さ，気分転換の役割，健全な気分転換方法について説明する  個人ワーク  ・生徒それぞれの普段の気分転換のリストアップさせる   説明  ・方法によって効果が異なることを説明する  ・実証研究から効果が確認された活動を紹介する   グループワーク  ・効果的な気分転換を参考に自分なりのリストを作成しグループで発表させる  まとめと宿題  ・教師によるレッスンのまとめ  　宿題：気分転換のリストをポスターにまとめる | |
| Lesson 3　「レジリエンスアルバム」 | | |
| ●授業の目標  ・「レジリエンス」に寄与する要因を理解する  ・自己のレジリエンス要因を確認する | | 授業で必要なリソース  ・ワークシート  ・詳細は書籍資料セッション9-12 |
| 導　入  展　開１  展　開２  展　開３  展　開４  まとめ | 「レジリエンスを鍛える」レジリエンスアルバムの紹介  ・レジリエンスに寄与する4つの要因（ソーシャルサポート，自尊感情，自己効力感，ポジティブ感情）について説明する  ・「レジリエンスアルバム」作成の説明をする  ワーク１：ソーシャルサポート  ・ワークシートに自分を支えている人の名前を書き込む  ワーク２：自分の強み  ・強みとその活用によるレジリエンスへの効果を説明する  ・強みについての少人数のグループワークを行う  ワーク３：困難からの回復  ・ワークシートを使用し失敗や挫折から立ち直った経験を振り返る  ・ペアで共有  ワーク４：ポジティブな回想写真  ・生徒が持参した自分の好きな写真を用い，コラージュを作成させる  まとめ  ・教師による学習のまとめ | |
| Lesson 4　逆境による成長 | | |
| ●授業の目標  ・心的外傷後成長（PTG）について学習し理解する | | 授業で必要なリソース  ・ワークシート  ・詳細は書籍資料セッション8 |
| 導　入  展　開１  展　開２  まとめ | PTSDとPTGの説明  ・逆境下の人間の自然なストレス反応とPTSDについて説明する  ・逆境体験からの成長可能性と心的外傷後成長(PTG)の概念を紹介する  ケーススタディ  ・ヴィクトール・フランクルの生涯について紹介する  ・彼の言葉や生涯からどんな教訓を学べるか，生徒と討議する  クラス討議  ・PTGの例を紹介し，これまでに自分がそのような場面を見聞きしたことがあるか尋ねる  ・クラス討議  まとめと宿題  ・教師によるレッスンのまとめ  宿題：PTGに類する事例を探し事例について検討するよう指示する | |
| Lesson 5　ネガティブスパイラルの解明 | | |
| ●授業の目標  ・状況，捉え方（認知）,　感情，行動，の関係性を知る  ・様々な「捉え方」とその反応を理解する | | 授業で必要なリソース  ・ワークシート  ・詳細は書籍資料セッション4,5 |
| 導　入  展　開１  展　開２  展　開３  まとめ | 「レジリエンス」に関連付け，ネガティブスパイラルが起こるメカニズムについて学ぶことを説明する  説明:「状況」「捉え方」「感情，自動反応」「行動」「結果，思い込み」の関係  ・同じ状況でも，人によって感情は違うことを確認する  ・「状況」と「感情，自動反応」の間に個々の「捉え方」があることを説明する  説明：「ネガティブな考え方（捉え方）」の紹介  ・ネガティブ・スパイラルの事例を紹介する  ・「捉え方」が果たす役割を検討する  ワーク：自分の捉え方のクセを発見  ・生徒に自分の経験を題材として「状況」，「感情，自動反応」，「ネガティブな捉え方」をワークシートに書き出させる  まとめおよび宿題  ・教師によるレッスンのまとめ | |
| Lesson 6　ネガティブスパイラルに挑戦 | | |
| ●授業の目標  ・自分の捉え方を客観視しその妥当性を検討する  ・捉え方を客観的に検討し，行動を選択取できる | | 授業で必要なリソース  ・ワークシート  ・詳細は書籍資料セッション6,7 |
| 導　入  展　開１  展　開２  展　開３  まとめ | ネガティブスパイラルへの挑戦の説明  ・L5を踏まえ，ポジティブな循環を検討することを説明する  クラス活動：ロールプレイを用いて捉え方を検討することを説明  ・ロールプレイのため３人（主人公，ネガティブな捉え方，第三者）の役を割り当てる  ・主人公の状況と捉え方，主人公の行動，思い込みについて説明する  ・主人公の捉え方の妥当性を客観的に検討し，生徒と討議する  説明：捉え方の代替案づくり  ・捉え方のポジティブな代替案づくりについて説明する  ・代替案を用いた結果，どのように感情や行動がポジティブに変化したかを説明する  ワーク：自分の捉え方に挑戦  ・L5を振り返り，ストレス体験，ネガティブ感情，その背景の捉え方を確認する  ・生徒に自分なりのポジティブな新しい捉え方を考えるワークを実施させる  まとめ  ・教師によるレッスンのまとめ | |

本プログラムはSPARKレジリエンスプログラム (Boniwell & Ryan, 2009)を日本の学校での実践を目的に短縮版として開発したものです。

本資料の内容を使用する際は下記を引用して下さい。

Kibe, C., Suzuki, M., Hirano, M., & Boniwell, I. (2020). Sensory processing sensitivity and culturally modified resilience education: Differential susceptibility in Japanese adolescents. PLOS One

日本語での授業案や資料の詳細は下記をご参照ください。

足立啓美・鈴木水季・久世浩司（2014）子どもの「逆境に負けない心」を育てる本　楽しいワークで身につく「レジリエンス」イローナ・ボニウェル（監修） 法研

ご不明な点，お問い合わせは下記までご連絡ください。

岐部 智恵子 Ph.D. Kibe.chieko@ocha.ac.jp
