# Supplementary material for: Sensory processing sensitivity and culturally modified resilience education: Differential susceptibility in Japanese adolescents
Source: PLoS One. 2020 Sep 14;15(9):e0239002. doi: 10.1371/journal.pone.0239002 (PMC7489542; doi:10.1371/journal.pone.0239002)
Supplement: S5 File — (DOCX) [file pone.0239002.s005.docx]

| **S5 File. Lesson Plan (English)** | | |
| --- | --- | --- |
| Lesson 1　What is “Resilience”? | | |
| ●Learning Objectives  ・Understand the construct of “resilience”  ・Define “resilience” in their own words | | Resources and materials  ・Worksheets  ・For details, see session 1,2 in Adachi et al. (2014) |
| Starter  Main 1  Main 2  Main 3  Plenary | Introduction of the “resilience” concept  Teacher to explain the main purpose of the program and the learning　objectives of the current lesson.  Students are given explanation on emotions, with emphasis on the nature and effects of negativity, which links to negative spiral of sequences.  Students to be given characteristics of resilience.  Activity 1  ・Promote students’ understanding of the concept   Activity 2  ・Students are given opportunity to share own version of definition of “resilience”.  Activity 3  ・Students are to draw their visual images of “resilience”  Conclusion  ・Teacher to summarize lesson.  Homework (optional) : Students are to find “resilience” example from YouTube. | |
| Lesson 2　Magic of Distraction | | |
| ● Learning Objectives  ・Understand the effectiveness of self-distraction when overwhelmed.  ・Identify effective strategies of self-distraction. | | Resources and materials  ・Worksheets  ・For details, see session 3 in Adachi et al. (2014) |
| Starter  Main１  Main ２  Main ３  Plenary | Introduction of the importance of effective self-distraction to prevent negative spiral  ・Students to review the effects of negative emotion and its spiral covered in L1  ・Students to learn the importance of recognition of emotion, the role of self-distraction, and the effective strategies to stop negative consequences  Activity 1  ・Students to list up their usual ways of self-distraction   Explain  Students to know the effective ways and ineffective ways of distractions with scientific reasonings. Teacher to share list of activities from empirical findings.   Group activity  Students to list up their own strategies based on the empirical findings, and present them in a small group.   Conclusion and Homework  Students to make a poster of their strategies listed in the group activity. | |
| Lesson 3　Resilience Muscle Training: Resilience Album | | |
| ●Learning Objectives  ・Understand the protective factors which contributes to ability to bounce back  ・Make own “resilience album” through activities | | Resources and materials  ・Worksheets  ・For details, see session 9-12 in Adachi et al. (2014) |
| Starter  Main１  Main ２  Main ３  Main ４  Plenary | Introduction of making “resilience album” to train resilience muscles  ・Teacher to explain four protective factors (social support, self-esteem, self-efficacy, positive emotion)  Activity 1: Social support  ・Using a worksheet, students to identify and name the people that they can count on  Activity ２：Finding my strengths  ・Teacher to introduce the concept of strengths and its function in relation to resilience  ・Students to undertake group activity to identify their own strengths.  Activity ３：I can bounce back  ・Students to reflect their own experiences of facing obstacles and bouncing back  ・Students to share their experiences with their peers.  Activity ４：Positive reflective photos  ・Students to bring their positive reflective photos to make a collage.  Conclusion  ・Teacher to summarize lesson. | |
| Lesson 4　Growing from adversity | | |
| **●Learning Objectives**  ・Learn about Post Traumatic Growth (PTG) | | Resources and materials  ・Visual aids from YouTube  ・For details, see session 8 in Adachi et al. (2014) |
| Starter  Main１  Main ２  Plenary | Introduction of PTSD and PTG  ・Students to know that natural human reactions in response to adversity, and PTSD as its effects.  ・Students to learn there are cases that people grow out of adversity and the concept of PTG.  Case study  ・Students to learn life of Viktor Frankl, and discuss what can be learned from his life and quotes.  Class discussion  ・Introduce some examples of PTG. Have a talk with the students around the theme.  Conclusion and homework  ・Teacher to reflect the lesson  ・Ask students to find some examples of PTG by researching various resources. | |
| Lesson 5　Understand the negative spiral | | |
| ●Learning Objectives  ・Learn the relationships among “situation”, “emotion/autopilot”, “reaction”, and “perception”  ・Understand multiple explanation styles of “perception” and their effects | | Resources and materials  ・Worksheet  ・For details, see session 4,5 in Adachi et al. (2014) |
| Starter  Main１  Main ２  Main 3  Plenary | Review the mechanism of “negative spiral” and the resilience skills  ・Teacher to explain the mechanism of negative spiral so the students understand the necessity to learn resilience skills  Introduction: Situation, Perception, Emotion/Autopilot, Reaction, Knowledge  ・Students to know the role of individual perception that induces various emotions even under the same circumstances  ・Teacher to explain the underlying perception induces emotion and reaction  Introduction：Negative perception  ・Teacher to tell a hypothetical example of negative sequential story that triggered by negative perception  ・Use metaphor to promote students’ understandings  Activity：Finding your own inner voice  ・Students to illustrate their own example of stressful event and identify each component of Situation, Perception, Emotion/Autopilot, Reaction, Knowledge.  Conclusion and homework  ・Teacher to reflect the lesson | |
| Lesson 6　Challenge your negative spiral | | |
| ●Learning Objectives  ・Objectify to validate their own perception  ・Evaluate their own perception then choose subsequence behavior | | Resources and materials  ・Worksheet  ・For details, see session 6,7 in Adachi et al. (2014) |
| Starter  Main１  Main ２  Main 3  Plenary | Introduction of the topic  ・Teacher to explain that they will learn the opposite mechanism of negative spiral  Class activity: Objectify the “perception” by role playing  ・Teacher to pick 3 students and assign them with the roles of “the main character”, “the negative inner voice”, and “the third person”.  ・Teacher to explain the situation, the negative inner voice (the perception), the behaviour, and the outcome knowledge.  ・Students to discuss to see the validity of the inner voice.  Explain: Alter the perception  Teacher to invite students to alter the perception from neutral/positive perspectives  Teacher to analyze how the perception changes after altering the wording  Activity：Challenge your own perception  ・Students to reflect L5 activity (”Finding your own inner voice”) and critically analyze the stressful situation, negative emotion, and the perception behind. Then try to alter the inner voice  Conclusion  ・Teacher to reflect the lesson | |

Notes.

This is a modified short version of SPARK resilience program (Boniwell & Ryan, 2009) adopted in Japan.

Should you wish to use this material, please acknowledge the source as shown below:

Kibe, C., Suzuki, M., Hirano, M., & Boniwell, I. (2020). Sensory processing sensitivity and culturally modified resilience education: Differential susceptibility in Japanese adolescents. PLOS One.

Detailed lesson plan and materials are available (Japanese only):

Adachi, H., Suzuki, M., & Kuze, K. (2014). The Resilience Building Program for Children and Teens ―with fun-filled resilience building activities―. I, Boniwell.(ed.). [Kodomo no “Gyakkyo ni makenai kokoro” wo sodateru hon]. Tokyo: Houken.

Contact for further information:

Chieko Kibe Ph.D. <kibe.chieko@ocha.ac.jp>
