# Supplementary material for: Sensory processing sensitivity and culturally modified resilience education: Differential susceptibility in Japanese adolescents
Source: PLoS One. 2020 Sep 14;15(9):e0239002. doi: 10.1371/journal.pone.0239002 (PMC7489542; doi:10.1371/journal.pone.0239002)
Supplement: S6 File — (DOCX) [file pone.0239002.s006.docx]

**S6 File. Items of Bidimensional Resilience Scale (BRS).**

| 1 | I think that things will work out on most occasions in any case. |
| --- | --- |
| 2 | I am good at preserving friendships since I was a child. |
| 3 | I understand my personality well. |
| 4 | I think that things will work out eventually even if I have no confidence. |
| 5 | It is good to be intimate with another person. |
| 6 | When I am faced with unpleasant situations, I try to gain something from those experiences. |
| 7 | I often fail to understand my own feelings or thoughts. (*) |
| 8 | I have enough stamina. |
| 9 | I value working hard. |
| 10 | I am good at detecting others’ feelings or changes in facial expressions. |
| 11 | I can handle difficult experiences well. |
| 12 | I can carry out decisions through to the end. |
| 13 | I treat everyone with consideration. |
| 14 | I think that I can manage to get over when faced with a difficult problem. |
| 15 | I am an outgoing person and have a wide circle of friends. |
| 16 | When misunderstandings arise with others, I willingly have more talks. |
| 17 | I understand how unpleasant things influence my feelings. |
| 18 | When I am faced with unpleasant situations, I try to gather information to solve the problem. |
| 19 | I can control my feelings even if there is a disagreement. |
| 20 | I think I have perseverance. |
| 21 | I am good at understanding others' ways of thinking. |

Notes. 1 = Strongly Disagree, 2 = Disagree, 3 = Neither Disagree nor Agree, 4 = Agree, 5 = Strongly Agree

*Question 7 is a reverse item.

This is an English translation from the original Japanese scale (Hirano, 2010)
